# Application of a Genetic Risk Score to Racially Diverse Type 1 Diabetes Populations Demonstrates the Need for Diversity in Risk-Modeling

**DOI:** 10.1038/s41598-018-22574-5

**Published:** 2018-03-14

**Authors:** Daniel J. Perry, Clive H. Wasserfall, Richard A. Oram, MacKenzie D. Williams, Amanda Posgai, Andrew B. Muir, Michael J. Haller, Desmond A. Schatz, Mark A. Wallet, Clayton E. Mathews, Mark A. Atkinson, Todd M. Brusko

**Affiliations:** 1 Departments of Pathology, Immunology, and Laboratory Medicine, College of Medicine, Gainesville, Florida USA; 2 Institute for Biomedical and Clinical Science, University of Exeter Medical School, Exeter, UK; 3 National Institute for Health Research, Exeter Clinical Research Facility, Exeter, UK; 4 Department of Pediatrics, Emory University, Atlanta, GA USA; 5 Department of Pediatrics, College of Medicine, Gainesville, Florida USA

## Abstract

Prior studies identified HLA class-II and 57 additional loci as contributors to genetic susceptibility for type 1 diabetes (T1D). We hypothesized that race and/or ethnicity would be contextually important for evaluating genetic risk markers previously identified from Caucasian/European cohorts. We determined the capacity for a combined genetic risk score (GRS) to discriminate disease-risk subgroups in a racially and ethnically diverse cohort from the southeastern U.S. including 637 T1D patients, 46 at-risk relatives having two or more T1D-related autoantibodies (≥2AAb^+^), 790 first-degree relatives (≤1AAb^+^), 68 second-degree relatives (≤1 AAb^+^), and 405 controls. GRS was higher among Caucasian T1D and at-risk subjects versus ≤ 1AAb^+^ relatives or controls (*P* < 0.001). GRS receiver operating characteristic AUC (AUROC) for T1D versus controls was 0.86 (*P* < 0.001, specificity = 73.9%, sensitivity = 83.3%) among all Caucasian subjects and 0.90 for Hispanic Caucasians (*P* < 0.001, specificity = 86.5%, sensitivity = 84.4%). Age-at-diagnosis negatively correlated with GRS (*P* < 0.001) and associated with HLA-DR3/DR4 diplotype. Conversely, GRS was less robust (AUROC = 0.75) and did not correlate with age-of-diagnosis for African Americans. Our findings confirm GRS should be further used in Caucasian populations to assign T1D risk for clinical trials designed for biomarker identification and development of personalized treatment strategies. We also highlight the need to develop a GRS model that accommodates racial diversity.

## Introduction

Type 1 diabetes susceptibility is largely controlled by HLA class-II genotype^1^, with modest contributions from at least 57 additional loci that confer varying degrees of disease protection or susceptibility^2^. Combinations of these alleles are thought to collectively determine an individual’s overall genetic risk, potentially resulting in heterogeneous disease presentation and etiology. The major role of HLA has been known for decades and is used as inclusion criteria for most studies of the disorder’s natural history. However, since HLA class-II is generally deemed to confer approximately half of the overall genetic risk^3–5^, it is likely that non-HLA risk loci could also be utilized to improve at-risk cohort stratification. Such measures of “total genetic risk” may be more effective at stratifying heterogeneous etiologies, for example, older progressors, who are less likely to develop multiple pre-clinical AAb and tend to have slower decline in C-peptide versus individuals with latent autoimmune diabetes in adults (LADA)^6,7^.

Large consortiums, such as the NIH-sponsored TrialNet, follow subjects at risk for type 1 diabetes and conduct intervention trials using composite scores that have factored in the presence of islet autoantibodies, family history, HLA risk haplotypes, as well as metabolic response markers^8^. These interventional efforts often aim to interdict the disease process at stage one, when there are already multiple autoantibodies present and a high likelihood of disease progression^9^. However, primary prevention trials aiming to avert the initiation of islet autoimmunity will likely require safe interventional efforts targeted to large population-based cohorts. Such cohorts can only be identified by genetics-based risk modeling near birth, given that autoantibody biomarkers that correspond to ongoing autoimmunity often occur early in life^10,11^. Consequently, the type 1 diabetes research community has developed increasingly informative genetic risk models based on genome wide association studies (GWAS) for risk prediction^12^.

The first attempts calculating overall type 1 diabetes genetic risk demonstrated that increased cumulative non-HLA risk alleles were associated with islet autoreactivity and disease onset, especially in the context of high-risk HLA^13,14^. These simple additive models only achieved area under the receiver operating characteristic curve (AUROC) of 0.66 for disease outcome. A major improvement was reported by Winkler *et al*. with their development of a multivariable logistic regression model to compute a type 1 diabetes genetic risk score (GRS)^15^. Using this model, they were able to discern patients from controls with AUROC of 0.87 to more accurately predict disease progression. In more recent works, Oram *et al*.^16^ and Patel *et al*.^17^ utilized a log-additive model^18^ to discriminate type 1 diabetes from type 2 diabetes patients (AUROC of 0.88) and subjects with monogenic forms of diabetes (AUROC of 0.87) from the Wellcome Trust Case Control Consortium (WTCCC) of British subjects with European Caucasian ancestry. Given the alarming increase in diabetes prevalence, particularly of type 2 diabetes in youth^19^ that may be difficult to discern from type 1 diabetes, there is a need to develop the GRS as a diagnostic tool. This requires examination of GRS in geographically distinct and demographically diverse patient populations with the potential for varying allele frequencies.

Our efforts reported herein expand previous analyses to southeastern United States populations, include considerations of race and ethnicity, and support the differential diagnostic utility of a GRS for clinical applications in type 1 diabetes. Such studies in distinct cohorts offer the potential to further refine the stratification of at-risk subjects and potentially elucidate type 1 diabetes subtypes. We examined several key features of the GRS, including its relationship with age of onset and the contribution of HLA class-II to that relationship, its utility in the context of subject race/ethnicity, and its capacity to aid in risk stratification.

## Results

### GRS effectively discerns type 1 diabetes patients and AAb^+^ individuals from controls and relatives within a Caucasian cohort

**Figure 1:**
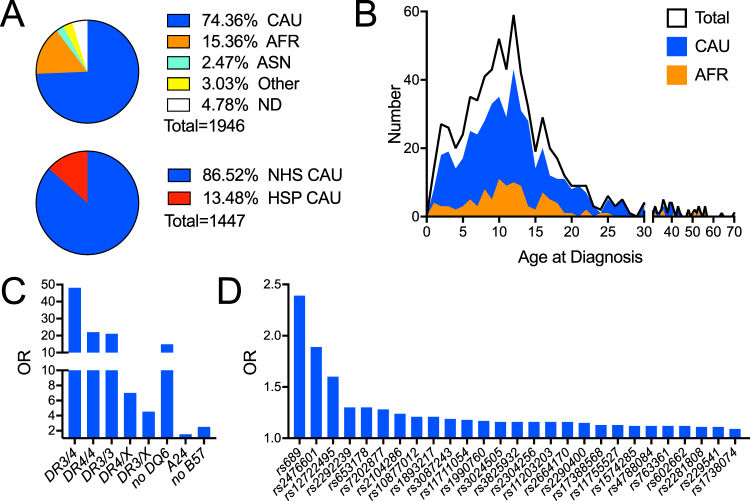
The University of Florida Diabetes Institute (UFDI) cohort demographics and loci used to calculate the Genetic Risk Score (GRS). (**A**) *Top panel*- Proportion of Caucasian (CAU), African American (AFR), Asian (ASN), Other (includes 0.26% Native American, 0.26 Pacific Islander, and 2.52% multiple races), and no data/not reported (ND). *Bottom panel*- Proportion of CAU that also self-reported as Hispanic (HSP) or non-Hispanic (NHS). (**B**) Age of diagnosis of the total, CAU, and AFR UFDI type 1 diabetes subjects. (**C**) Odds ratios (OR) for HLA diplotypes (DR3/4, DR4/4, DR3/3, DR4/X, and DR3/X) and haplotypes (non-DQ6, A24, and non-B57) used to compute the GRS. (**D**) OR for non-HLA loci.

Until now, type 1 diabetes GRS regression models put forth by Oram *et al*. and Patel *et al*.^16,17^ have only been tested and validated in European Caucasian cohorts^15–17^. We sought to determine the efficacy of a similar GRS, calculated as previously described^16,17^, in our regional southeastern U.S. cohort comprised of type 1 diabetes patients [n = 637, age (years) median (interquartile range) 15.50 (11.67–19.75)], first-degree relatives [≤1AAb^+^, n = 790, age 20.75 (11.29–40.42)], second-degree relatives [≤1AAb^+^, n = 68, age 26.79 (12.33–45.02)], at-risk relatives (≥2AAb^+^, n = 46, age 15.33 (10.33–33.83)], and controls [n = 405, age 23.92 (16.42–33.25)] of various racial and ethnic backgrounds, including Caucasian, African, and Asian Americans (Fig. 1A top and 1B; Supplemental Tables 1 and 2). A genotyping panel composed of HLA imputing SNPs (Fig. 1C) and putatively identified non-HLA SNPs associated with disease risk (Fig. 1D) was utilized to compute a log-additive type 1 diabetes GRS. Since the HLA locus does not fit this log-additive model^1,20^, we used published ORs for imputed HLA diplotypes (Supplemental Table 3).

**Figure 2:**
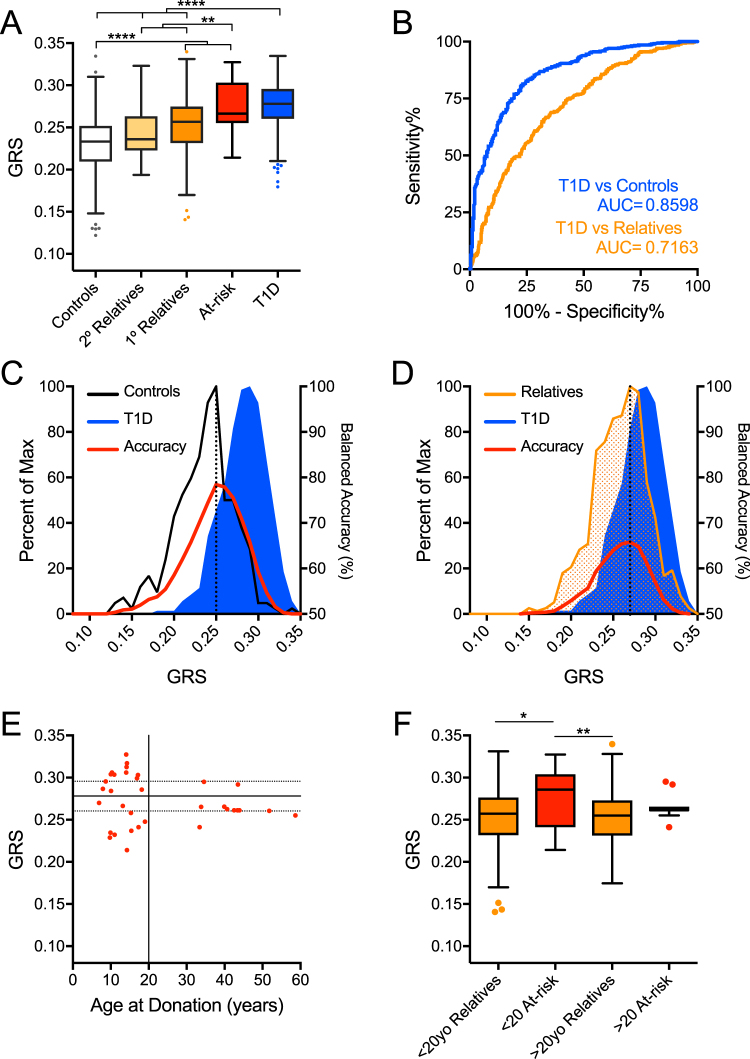
The Genetic Risk Score (GRS) can discriminate Caucasian subjects with type 1 diabetes and high-risk relatives from controls and lower-risk relatives. (**A**) GRS was significantly higher among Caucasian type 1 diabetes patients (T1D, n = 478) and at-risk relatives (n = 35) compared to controls (n = 290), second-degree relatives (2° Relatives, n = 33), and first-degree relatives (1° Relatives, n = 611). (**B**) Receiver operating characteristic (ROC) curve shows that the GRS significantly discriminates type 1 diabetes patients from control subjects (T1D vs Controls) with 83.3% sensitivity yielding 73.9% specificity (area under curve (AUC) = 0.8598) and, to a lesser degree, type 1 diabetes patients from first-degree relatives (T1D vs Relatives) with 67.4% sensitivity yielding 65.0% specificity (AUC = 0.7163). (**C**) Classifying subjects as T1D or control. Peak balanced accuracy was determined to be 78.95% at a GRS of 0.251. (**D**) Classifying subjects as T1D or relatives. Peak balanced accuracy was 66.70% at a GRS of 0.267. (**E**) GRS of At-risk subjects (≥2AAb^+^) vs age at donation. The 75^th^ (upper dotted), 50^th^ (solid), and 25^th^ (lower dotted) centile lines of the T1D GRS are shown for reference. (**F**) Comparison of GRS of young (<20 years old) At-risk subjects to aged (>20) At-risk, young first-degree relatives, aged first-degree relatives. Kruskal-Wallis ANOVA with Dunn’s posttest *P < 0.05, **P < 0.01, ****P < 0.0001.

We began with the Caucasian subject set from the UFDI cohort, which may be genetically distinct from the European cohorts. Notably, 13.49% the Caucasian subjects reported Hispanic/Latino ethnicity (Fig. 1A bottom). The GRS was significantly higher for type 1 diabetes patients (n = 478, mean ± SD 0.277 ± 0.03, *P* < 0.0001 for all comparisons) compared to controls (n = 290, 0.231 ± 0.03), second-degree relatives (n = 33, 0.244 ± 0.03) and first-degree relatives (n = 611, 0.253 ± 0.03) (Fig. 2A). Anticipated dilution of risk in first- and second-degree relatives was also evident. Notably, at-risk relatives (≥2 AAb^+^) had a GRS (0.274 ± 0.03, *P* > 0.9999) similar to type 1 diabetes patients (Fig. 2A). ROC analysis demonstrated the GRS significantly discriminated between type 1 diabetes patients and control subjects, providing 73.9% specificity with 83.3% sensitivity for accurately detecting type 1 diabetes by GRS alone (AUROC = 0.86, Fig. 2B). As expected, it was less effective at discriminating type 1 diabetes from first-degree relatives (65.0% specificity, 67.4% sensitivity, AUROC = 0.72, Fig. 2B). To examine the utility of this model in classifying individuals as type 1 diabetes subjects, controls, or relatives, we calculated the balanced accuracy across the distribution of the GRS range. We found that 0.251 was the optimal threshold to classify type 1 diabetes and control subjects in this GRS model, with accuracy of 79.0% (Fig. 2C) and that a GRS threshold of 0.267 yielded accuracy of 66.7% for classifying type 1 diabetes subjects from relatives (Fig. 2D).

Multiple AAb^+^ subjects under 20 years of age have the highest probability of progressing to disease and may already have subclinical type 1 diabetes^21^. It is less clear whether older multiple AAb^+^ individuals will progress to disease. Consistent with this, at-risk subjects under 20 years of age in our cohort had a GRS (0.277 ± 0.03) identical to the type 1 diabetes patients’ GRS. We observed that 56.5% of at-risk subjects under 20 years of age had GRS above the UFDI type 1 diabetes patient cohort 50^th^ centile (Fig. 2E, solid horizontal line) compared to only 22.2% of the at-risk subjects above 20 years of age. Further, for subjects <20 years of age, GRS of at-risk subjects was significantly higher than relatives, while for >20 year olds, GRS of at-risk subjects was similar to relatives (Fig. 2F).

**Figure 3:**
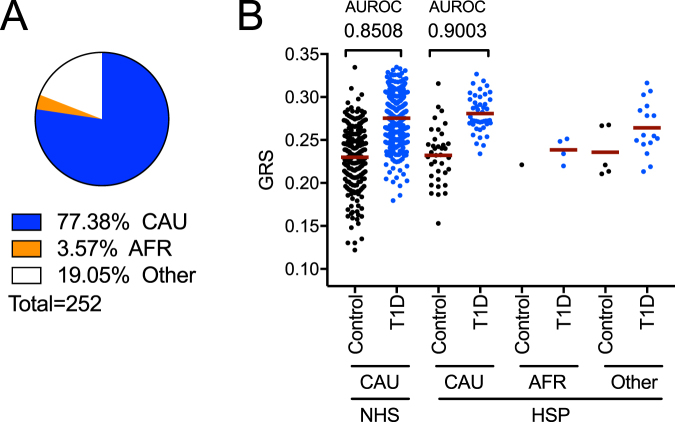
GRS assessment of Hispanic ethnicity by race. (**A**) Proportion of CAU, AFR and Other (multiple or not reported) in subjects that self-reported as Hispanic (HSP) ethnicity. (**B**) Comparison of HSP ethnicity subjects by race to CAU non-Hispanic (NHS) subjects indicates that GRS discriminates patients from control HSP CAU subjects as well as it does for NHS CAU.

In addition to reporting race, subjects also reported ethnicity as Hispanic/Latino or non-Hispanic/Latino. Hispanic individuals represent a genetically diverse population with mostly European, Native American, and African admixtures^22,23^. Since this GRS was modeled from Caucasian American and European genetic frequencies, we sought to investigate its accuracy on this diverse population. 12.9% (n = 252) of the total UFDI cohort (all races) self-reported as Hispanic/Latino. Within these, 77.38% (n = 195) reported as Caucasian, 3.57% (n = 9) as African American, and 19.05% (n = 48) as Other (multiple race or not reported, Fig. 3A; Supplemental Table 2). We found that the GRS discriminated Hispanic/Latino Caucasian patients (n = 45, 0.281 ± 0.02) from Hispanic/Latino Caucasian controls (n = 37, 0.232 ± 0.03; AUROC = 0.90) with efficacy comparable to non-Hispanic/Latino Caucasian patients (n = 433, 0.275 ± 0.03) from non-Hispanic/Latino Caucasian controls (n = 253, 0.230 ± 0.04; AUROC = 0.85) (Fig. 3B). Among Caucasian controls, mean GRS was similar for Hispanic/Latino and non-Hispanic/Latino cohorts (Mann-Whitney *P* = 0.981). Moreover, the GRS of this U.S.-derived Caucasian cohort, which includes Hispanic/Latino Caucasian subjects, was comparable to the GRS of a European-derived Caucasian cohort (Supplemental Table 4)^16,17,24^. The Hispanic African American and Hispanic Other cohorts are shown for comparison, but were not sufficiently powered for analysis (Fig. 3B).

### Higher GRS associates with a younger age at diagnosis in Caucasian subjects

**Figure 4:**
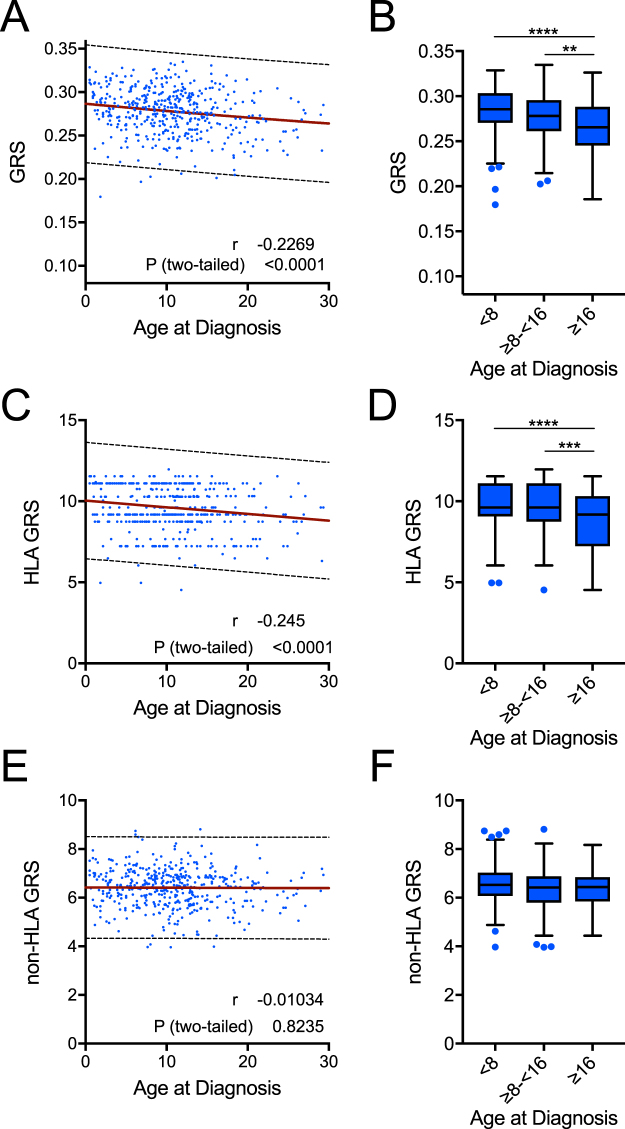
HLA risk imparts a genetic association with age of disease onset. (**A**) The genetic risk score (GRS) was significantly and inversely correlated with age at diagnosis (linear regression analysis and Pearson correlation coefficient, *P* < 0.001, r = −0.227). (**B**) GRS was significantly different in patients when grouped into under 8, 8–16, and over 16 years old at diagnosis (Kruskal-Wallis ANOVA with Dunn’s posttest **P < 0.01, ****P < 0.0001). (**C, D**) The HLA-only GRS imparted a similar association with age at diagnosis as the full score (C: linear regression analysis and Pearson correlation coefficient, *P* < 0.001, r = −0.245; D: Kruskal-Wallis ANOVA with Dunn’s posttest *** *P* < 0.001, ****P < 0.0001). (**E**, **F**) The non-HLA GRS did not correlate with age at diagnosis (E: linear regression analysis and Pearson correlation coefficient, *P* > 0.05, r = −0.010; F: Kruskal-Wallis ANOVA with Dunn’s posttest *P* > 0.05). The 99% probability bands for linear regressions are depicted as dotted lines.

We next addressed whether GRS was associated with type 1 diabetes age of onset. Indeed, among Caucasian type 1 diabetes subjects, we observed a significant negative correlation between GRS and age of diagnosis (Pearson’s correlation r = −0.23, *P* < 0.0001, Fig. 4A). Subjects diagnosed after age 16 had lower GRS than those diagnosed from 8–16 years of age and those diagnosed under age 8 (Fig. 4B), suggesting that a higher GRS may predict earlier disease onset. Prior studies noted the HLA association with earlier onset of disease; however, the contribution of the non-HLA component of risk was not clear^25–27^. Our data clearly demonstrated the majority, if not all, of this negative age association was conferred by the HLA risk component. When the non-HLA loci were removed from the GRS calculation, the negative correlation with age (r = −0.25, *P* < 0.0001) was virtually the same as the full GRS (Fig. 4C,D). Conversely, when HLA was removed from the calculation, no association with age at diagnosis was observed (Fig. 4E,F).

**Figure 5:**
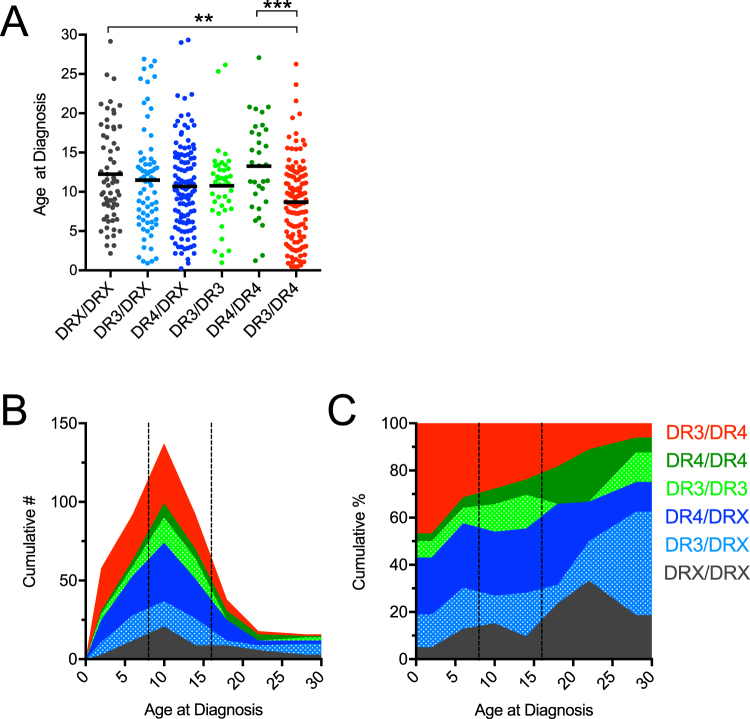
HLA versus Age at Diagnosis. (**A**) Patients with the highest risk HLA-DR3/DR4 had a lower age at diagnosis. Kruskal-Wallis ANOVA with Dunn’s posttest **P < 0.01, ***P < 0.001. (**B**) Stacked histogram depicting the cumulative number of patients grouped by HLA type versus their ages at diagnosis (4 year binned). (**C**) Stacked histogram depicting the cumulative percent of patients grouped by HLA type versus their ages at diagnosis (4 year binned). (**B,C**) HLA-type is indicated by color as shown within the figure, and 8-year and 16-year age cutoffs are indicated by dashed lines.

We next sought to determine which HLA diplotypes may be affecting the age at diagnosis. High-risk HLA-DR3-DQ2 (simplified to DR3) and HLA-DR4-DQ8 (simplified to DR4) were imputed and subjects were categorized into six diplotypes in combination with lower-risk HLA (collectively denoted as DRX): DR3/DR4, DR4/DR4, DR3/DR3, DR4/DRX, DR3/DRX, and DRX/DRX. We observed the known contribution of HLA-DR3/DR4 to earlier clinical onset^28^, as well as a significant difference in age of diagnosis between HLA-DR3/DR4 and HLA-DR4/DR4 subjects (Fig. 5A). Distributions of numbers (Fig. 5B) and percentages (Fig. 5C) of age of diagnosis stacked by HLA risk diplotypes illustrate the skewing of HLA-DR3/DR4 individuals to earlier diagnoses. To quantify this observation, we calculated the proportion of patients diagnosed prior to 8 years of age, from 8–16 years of age, and older than 16 years of age for each of the six HLA categories (Table 1). We found that the proportion of patients with HLA-DR3/DR4 diagnosed before age 8 (44.4%) was 5.6 times greater (*P* < 0.01) than those diagnosed after age 16 (7.9%), while the proportion for the other five HLA categories diagnosed before age 8 (28.9%) was only 1.5 times greater than those diagnosed after age 16 (19.1%). Conversely, significantly more patients with HLA-DR4/DR4 (*P* < 0.01) and DRX/DRX (*P* < 0.05) diplotypes were diagnosed after age 16. Interestingly, HLA-DR3/DR3 patients were more likely to be diagnosed between age 8 and 16 (*P* < 0.01, Table 1). These results suggest that contribution of high-risk HLA-DR3 and HLA-DR4 haplotypes to age of clinical onset may be more nuanced than previously reported^25–28^.

**Table 1:** HLA versus Age at Diagnosis. The proportion of type 1 diabetes subjects diagnosed at age <8, age ≥8 to <16, and age ≥16 years within each HLA category is reported as number (N) and as odds ratio (OR). The OR is calculated for age groups within each HLA diplotype (i.e., the OR of DR3/DR4 patients being diagnosed under 8 years of age is 1.97 as compared to DR3/DR4 patients diagnosed over 8 years of age). Fisher’s exact test was used to determine if age at diagnosis was significantly different for each HLA type. ^†^*P* < 0.05, ^‡^*P* < 0.01.

Age at Diagnosis	DRX/DRX		DR3/DRX		DR4/DRX		DR3/DR3		DR4/DR4		DR3/DR4	
	N	(OR)	N	(OR)	N	(OR)	N	(OR)	N	(OR)	N	(OR)
<8	15	(0.59)	24	(1.06)	39	(0.97)	10	(0.62)	6	(0.44)	56	(1.97^‡^)
8–16	30	(0.86)	33	(0.84)	62	(1.07)	29	(2.54^‡^)	15	(0.85)	60	(0.84)
>16	18	(2.47^‡^)	13	(1.24)	18	(0.92)	2	(0.25^†^)	11	(3.07^‡^)	10	(0.37^‡^)

Oram and Patel initially used a similar GRS model as a tool to assist in the differential diagnoses of early onset type 2 diabetes and monogenic forms of diabetes from type 1 diabetes^16,17^. Within the UFDI cohort, we identified five type 1 diabetes subjects with GRS values below the 99^th^ percentile prediction band of GRS versus age at diagnosis (Fig. 4A). Initially, we observed 3 additional type 1 diabetes subjects with exceptionally low GRS that were all AAb^-^ at onset (data not shown). Clinical follow-up revealed that two of these subjects (subject 1: age at diagnosis (yrs) = 10, BMI = 28.0, GRS = 0.118; subject 2: age at diagnosis = 15, BMI = 19.3, GRS = 0.153) were undergoing MODY testing (awaiting patient compliance), and the third (age at diagnosis = 16, BMI = 42.0, GRS = 0.184) has been re-diagnosed as having type 2 diabetes. These results support the utility of GRS in aiding in the differential diagnosis of diabetes forms when used in combination with standard clinical assessments and AAb detection.

### Current GRS models are less robust for assessing type 1 diabetes risk in U.S. racial minority groups

We next examined the utility of GRS to discriminate type 1 diabetes subjects from controls or relatives within the Asian American and African American (includes Hispanic/Latino African Americans, Supplemental Table 2) subsets of the UFDI cohort. This notion emanates from previous HLA associations in African American type 1 diabetes subjects^29^, in addition to clear alterations in the allele frequencies of racial groups for the putative risk loci reported in the 1000 Genomes project^30^. Similar to Caucasian subjects (Fig. 2), GRS was significantly higher in Asian American type 1 diabetes subjects compared to controls (Fig. S1A). GRS appeared to accurately discriminate type 1 diabetes patients from controls (AUROC = 0.92; *P* = 0.0002) and from relatives with (AUROC = 0.86; *P* = 0.04) (Fig. S1B), although this cohort is insufficiently powered to draw conclusive results at a population scale (Supplemental Table 1). Additionally, no multiple AAb^+^ at-risk Asian American subjects were enrolled in this study; hence, there is a need to validate these findings in a larger cohort.

**Figure 6:**
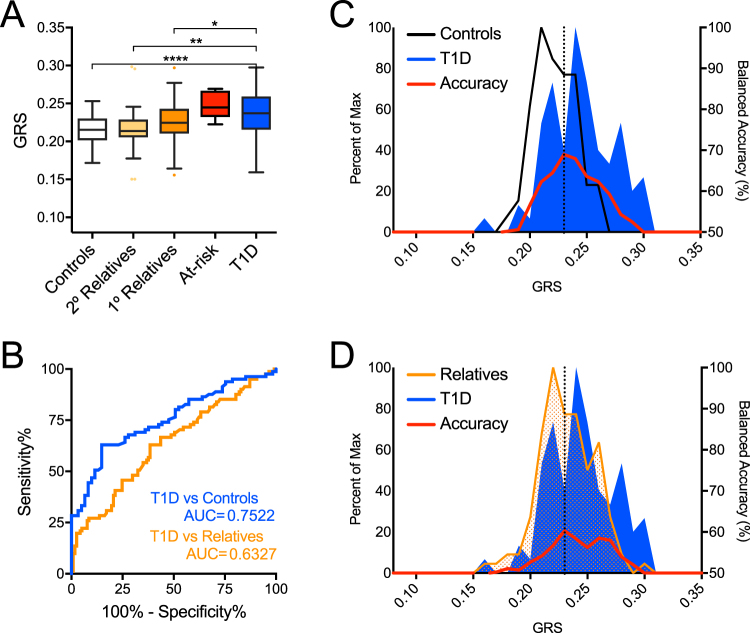
GRS poorly discriminates African American (AFR) subjects with type 1 diabetes and high-risk relatives from controls and lower-risk relatives. (**A**) GRS was higher among type 1 diabetes patients (T1D, n = 84) and at-risk relatives (n = 6) compared to controls (n = 63), second-degree relatives (2° Relatives, n = 28), and first-degree relatives (1° Relatives, n = 118). Kruskal-Wallis ANOVA with Dunn’s posttest *P < 0.05, **P < 0.01, ****P < 0.0001. (**B**) Receiver operating characteristic (ROC) curve shows that the GRS discriminates type 1 diabetes patients from control subjects (T1D vs Controls) with 62.96% sensitivity yielding 85.25% specificity (area under curve (AUC) = 0.7522) and type 1 diabetes patients from first-degree relatives (T1D vs Relatives) with 62.96% sensitivity yielding 61.54% specificity (AUC = 0.6327). (**C**) Classifying subjects as T1D or Control. Peak balanced accuracy was determined to be 68.98% at a GRS of 0.233. (**D**) Classifying subjects as T1D or relatives. Peak balanced accuracy was 60.39% at a GRS of 0.233.

Once again, GRS was significantly higher in African American type 1 diabetes patients (n = 84) compared to controls (n = 63) as well as relatives (n = 118), but the study was not sufficiently powered to detect significant differences from multiple AAb^+^ at-risk African American subjects (n = 6, Fig. 6A; Supplemental Table 1). Within the African American cohort, we found GRS was less robust in discerning type 1 diabetes patients from controls (63.0% sensitivity, 85.3% specificity, AUROC = 0.75) or from first-degree relatives (63.0% sensitivity, 61.5% specificity, AUROC = 0.63) (Fig. 6B). Peak balanced accuracy was 68.98% at GRS = 0.233 for classifying African American subjects as type 1 diabetes patients or controls and 60.30% at GRS = 0.233 for classifying subjects as patients or relatives (Fig. 6C,D). Additionally, the HLA-mediated association between GRS and age of diagnosis observed in Caucasian patients was lost in the African American cohort (Figs S2–S3; Supplemental Table 5). HLA associated with the highest risk in Caucasians were detected in lower frequencies in African Americans, where the three highest risk HLA (HLA-DR3/DR4, -DR4/DR4, and -DR3/DR3) were only detected in African American patients and not in controls (Fig. S3; Table 2). Importantly, the SNP array utilized herein did not impute the African American-derived HLA haplotypes shown to confer type 1 diabetes risk or protection^29^. Though only modestly powered, several non-HLA alleles tested for GRS did not confer risk in African Americans to the same degree as in Caucasians (Table 2). Notable risk differences were observed for three SNPs tested herein: *SH2B3* conferred higher risk in UFDI African Americans (OR = 2.93 [95% CI, 1.22–7.03], *P* = 0.013) than UFDI Caucasians (OR = 1.30 [1.06–1.59], *P* = 0.014); *CTRB1/2* was protective in African Americans, though it did not achieve significance (OR = 0.55 [0.97–3.44], *P* = 0.08) in contrast to Caucasians where *CTRB1/2* was clearly associated with risk (OR = 1.56 [95% CI, 1.12–2.17], *P* = 0.008); *GAB3* only conferred risk in African Americans (OR = 1.82 [1.09–3.04], *P* = 0.028) and not in Caucasians (OR = 0.89 [0.88–1.44], *P* > 0.1) (Table 2).

**Table 2:** Comparison of the genetic risk score (GRS) loci within the University of Florida Diabetes Institute (UFDI) Caucasian (CAU) and African American (AFR) cohorts. For each single nucleotide polymorphism (SNP) examined, the locus in the human genome, associated candidate gene(s), and allele, haplotype, or diplotype measured are listed along with frequency and odds ratios (OR) in the type 1 diabetes (T1D) and control cohorts from the UFDI CAU and AFR subject cohorts. The statistics shown are for the published risk alleles (as opposed to the minor alleles) as referenced in Supplemental Table 3. ^†^Statistics for protective HLA DR15-DQ6 and B57 are shown for the non-DR15-DQ6 and non-B57 risk haplotypes.

SNP	Locus	Candidate Gene(s)	Genotype Measured	UFDI CAU Cohort					UFDI AFR Cohort				
				T1D Frequency	Control Frequency	OR	95% CI	p-value	T1D Frequency	Control Frequency	OR	95% CI	p-value
rs2187668 rs7454108	6p21.32	*HLA DR3-DQ2 HLA DR4-DQ8*	DR3/4	0.2636	0.0347	9.95	5.13–19.31	2.72 × 10^-18^	0.0833	0.0000	—	—	2.00 × 10^-2^
			DR4/4	0.0690	0.0313	2.30	1.08–4.88	3.21 × 10^-2^	0.0238	0.0000	—	—	0.51
			DR3/3	0.0962	0.0035	30.56	4.19–222.85	3.61 × 10^-9^	0.0595	0.0000	—	—	0.07
			DR4/X	0.2573	0.1701	1.69	1.17–2.45	5.52 × 10^-3^	0.1905	0.0635	3.47	1.1–10.96	2.97 × 10^-2^
			DR3/X	0.1569	0.1979	0.75	0.52–1.1	0.17	0.2262	0.1587	1.55	0.66–3.61	0.40
			DRX/X	0.1569	0.5625	0.14	0.1–0.2	1.90 × 10^-31^	0.4167	0.7778	0.20	0.1–0.43	1.15 × 10^-5^
rs3129889	6p21.32	*HLA DR15-DQ6* ^†^	X^†^	0.9822	0.8802	7.52	4.37–12.92	1.35 × 10^−16^	0.9940	0.9921	1.32	0.08–21.31	1.00
rs1264813	6p21.32	*HLA A24*	A24	0.8800	0.9100	0.72	0.51–1.02	7.54 × 10^–2^	0.9464	0.9524	0.88	0.31 − 2.55	1.00
rs2395029	6p21.32	*HLA B57* ^†^	X^†^	0.9927	0.9790	2.91	1.14–7.42	2.93 × 10^−2^	1.0000	0.9921	—	—	0.43
rs2476601	1p13.2	*PTPN22*	A	0.1542	0.0734	2.30	1.61–3.28	1.72 × 10^−6^	0.0179	0.0154	1.16	0.19–7.07	1.00
rs3024505	1q32.1	*IL10*	G	0.8724	0.8616	1.10	0.81–1.49	0.54	0.9458	0.9385	1.14	0.43–3.05	0.81
rs1990760	2q24.2	*IFIH1*	T	0.6330	0.6211	1.05	0.85–1.3	0.66	0.2143	0.2109	1.02	0.58–1.81	1.00
rs3087243	2q33.2	*CTLA4*	G	0.5866	0.5533	1.15	0.93–1.41	0.20	0.7927	0.7000	1.64	0.96–2.79	0.08
rs11711054	3p21.31	*CCR5*	A	0.6542	0.6862	0.86	0.93–1.44	0.20	0.8095	0.7846	1.17	0.66–2.06	0.66
rs17388568	4q27	*ADAD1 IL2 IL21*	A	0.2587	0.2491	1.05	0.83–1.33	0.72	0.0783	0.0769	1.02	0.43–2.41	1.00
rs11755527	6q15	*BACH2*	G	0.4508	0.4659	0.94	0.87–1.3	0.56	0.2262	0.2000	1.17	0.67–2.05	0.67
rs1738074	6q25.3	*TAGAP*	C	0.5806	0.5531	1.12	0.91–1.38	0.29	0.3095	0.2769	1.17	0.71–1.94	0.61
rs1574285	9p24.2	*GLIS3*	G	0.4775	0.4539	1.10	0.9–1.35	0.37	0.5361	0.5625	0.90	0.7–1.77	0.72
rs12722495	10p15.1	*IL2RA*	T	0.9146	0.8716	1.58	1.13–2.2	9.01 × 10^−3^	0.9819	0.9692	1.72	0.38–7.85	0.70
rs2104286	10p15.1	*IL2RA*	T	0.8031	0.6962	1.78	1.4–2.25	2.12 × 10^−6^	0.9583	0.9231	1.92	0.71–5.18	0.22
rs689	11p15.5	*INS INS-IGF2 TH*	T	0.8357	0.6843	2.35	1.84–2.99	5.23 × 10^−12^	0.3598	0.2698	1.52	0.92–2.52	0.13
rs2292239	12q13.2	*ERBB3*	T	0.3792	0.3287	1.25	1–1.55	4.84 × 10^−2^	0.4286	0.3889	1.18	0.74–1.89	0.55
rs10877012	12q14.1	*CYP27B1*	G	0.7118	0.6980	1.07	0.85–1.34	0.57	0.8750	0.8462	1.27	0.66–2.46	0.50
rs653178	12q24.12	*ATXN2 SH2B3 NAA25*	C	0.5254	0.4608	1.30	1.06–1.59	1.42 × 10^−2^	0.1429	0.0538	2.93	1.22–7.03	1.30 × 10^−2^
rs3825932	15q25.1	*CTSH*	C	0.6697	0.6695	1.00	0.8–1.24	1.00	0.1964	0.2231	0.85	0.67–2.06	0.67
rs4788084	16p11.2	*NUPR1 IL27*	C	0.6392	0.5773	1.30	1.05–1.6	1.56 × 10^−2^	0.7711	0.7462	1.15	0.67–1.96	0.68
rs7202877	16q23.1	*CTRB2 CTRB1*	G	0.1395	0.0942	1.56	1.12–2.17	8.48 × 10^−3^	0.1205	0.2000	0.55	0.97–3.44	0.08
rs2290400	17q12	*GSDMB ORMDL3*	C	0.5071	0.4846	1.09	0.89–1.34	0.40	0.4643	0.5078	0.84	0.75–1.89	0.48
rs7221109	17q21.2	*CCR7 SMARCE1*	T	0.3686	0.3564	1.05	0.85–1.31	0.66	0.1488	0.1587	0.93	0.57–2.05	0.87
rs1893217	18p11.21	*PTPN2*	G	0.1955	0.1598	1.28	0.97–1.68	0.08	0.1098	0.1077	1.02	0.49–2.14	1.00
rs763361	18q22.2	*CD226*	T	0.5346	0.4691	1.30	1.06–1.6	1.39 × 10^−2^	0.6905	0.7540	0.73	0.82–2.31	0.24
rs2304256	19p13.2	*TYK2*	C	0.7127	0.6986	1.07	0.85–1.34	0.56	0.8855	0.8516	1.35	0.68–2.67	0.48
rs602662	19q13.33	*FUT2*	A	0.5010	0.5034	0.99	0.82–1.24	0.96	0.5714	0.5000	1.33	0.84–2.11	0.24
rs2281808	20p13	*SIRPG SIRPB1*	C	0.6870	0.6901	0.99	0.81–1.27	0.91	0.8012	0.7538	1.32	0.76–2.29	0.40
rs11203203	21q22.3	*UBASH3A*	A	0.3699	0.3265	1.21	0.98–1.5	0.09	0.1627	0.1385	1.21	0.63–2.31	0.63
rs229541	22q12.3	*RAC2*	G	0.5645	0.5295	1.15	0.94–1.42	0.19	0.3512	0.3385	1.06	0.65–1.71	0.90
rs2664170	Xq28	*GAB3*	G	0.3426	0.3695	0.89	0.88–1.44	0.37	0.5882	0.4393	1.82	1.09–3.04	2.77 × 10^−2^

## Discussion

Focused genetic testing is relatively inexpensive, non-invasive, and may be scaled for population screening efforts. The implementation of such tests may be useful for refining efforts to identify subjects who would benefit from more costly AAb and interventional screening efforts that may need to be repeated over time. Given that type 1 diabetes has known genetic components conferring susceptibility, several models designed to stratify subjects as high- and low-risk have been developed in recent years. One model was recently shown to assist in the differential diagnosis of type 1 diabetes from early-onset type 2 diabetes and from monogenic diabetes^16,17^. We emulated this model to assess its capacity to stratify subgroups (i.e., controls, low- and high-risk relatives, type 1 diabetes patients) using cumulative genetic risk in our regional cross-sectional cohort. While the Oram *et al*. and the Winkler *et al*. models both report similar AUROC, we chose to use the Oram *et al*. model because it employs published, accessible OR to set weights for T1D risk loci. Using this approach, we were able to segregate Caucasian type 1 diabetes subjects from controls with 79.0% accuracy and an AUROC = 0.86. As a comparison, in a European Caucasian cohort roughly 7 times larger, Winkler *et al*. reported a patient versus control ROC AUC = 0.87^31^. Of note, GRS of our UFDI cohort were comparable to those previously scored for the WTCCC^16,17,24^ (Supplemental Table 5**)**. As expected, the GRS was higher in subjects with type 1 diabetes compared to first-degree relatives, second-degree relatives, and controls. Most importantly, the GRS was also significantly higher in relatives at the highest-risk for disease development (≥2 AAb^+^) compared lower-risk relatives (≤1 AAb^+^); this was the case even in subjects under 20 years of age (0.277 ± 0.03). We note that this current study did not measure anti-insulin autoantibodies, which may have affected subject assignment as ≤1 AAb^+^ relatives and ≥2AAb^+^ at-risk relatives. These data support the notion that genotyping a limited number of selected SNPs allows for the identification of subjects at elevated-risk for developing disease. This notion has important implications for GRS use for subject enrollment into mechanistic and natural history studies of type 1 diabetes. It also highlights potential for large-scale population screening efforts for clinical diagnostics, particularly as per sample genotyping costs decline over time. We acknowledge that our genotyping is by no means comprehensive, and the potential may exist to improve prediction and ROC values as additional validated loci and causative SNPs are defined. This may be particularly true regarding ROC for type 1 diabetes subjects versus relatives sharing an appreciable portion of the genome. Ultimately, long-term longitudinal studies such as TEDDY, DAISY, TrialNet Natural History Study, and BABYDIAB will be most informative for such analyses^14,15,32^.

Genetic screening may not only identify high-risk individuals, but may also indicate appropriate ages to implement other screening regimens, such as AAb testing. We found a significant negative correlation between GRS and age of type 1 diabetes diagnosis, which was nearly completely accounted for by HLA diplotype. While the highest-risk HLA-DR3/DR4 diplotype was associated with the earliest age of diagnosis, as has been previously shown^25–27^, diagnosis occurred significantly later and coincided with puberty in subjects carrying the HLA-DR3/DR3 diplotype. The genes that comprise GRS account for a major proportion of the heritability of type 1 diabetes but explain much less of the variation of the heterogeneity of age of diagnosis. Improvements in this latter capacity may require better powered approaches or GWAS designed to identify genetic associations of type 1 diabetes characteristics (such as diagnosis age^33^ and rate of β-cell decline), which may be distinct from the variants associated with disease development.

The SEARCH for Diabetes in Youth Study recently reported that type 1 diabetes prevalence was 1 in 392 for Caucasian Americans under 20 years of age, 1 in 617 for African Americans, and 1 in 1667 for Asian Americans^34,35^. Currently, the vast majority of type 1 diabetes genetics studies are limited to Caucasian cohorts. However, the figures above imply that for African Americans, type 1 diabetes prevalence is almost 2/3 that of Caucasian Americans, while for Asian Americans it is almost 1/4. Thus, closer examinations of type 1 diabetes genetics within these underrepresented racial minorities in the U.S. must be performed. A limitation of the current study was our reliance on self-reported race and ethnicity, as our SNP array lacked informative ancestral markers commonly utilized in high density genome-wide arrays for imputing and assigning race and ethnicity. Nevertheless, our analysis indicated that GRS could discriminate type 1 diabetes subjects from controls in a small cohort of subjects identifying as Asian American, but larger studies are need to validate and extend these findings. For African Americans however, GRS was less effective in discerning type 1 diabetes subjects from controls, and the association between a higher GRS with early disease onset was lost. These observations are likely related to known differences in HLA-conferred disease risk or protection in the context of race^27,29,36^. Thus, GRS models suitable for African Americans would likely need to impute these haplotypes. Additionally, the current set of type 1 diabetes risk loci, which were identified in predominantly Caucasian cohorts, may be less effective for assessing risk in non-Caucasian individuals. These findings underscore the need to perform type 1 diabetes incidence studies and GWAS in non-Caucasian groups, enabling development of a GRS model that accounts for heterogeneity in populations.

Type 1 diabetes risk-loci that significantly predict disease in African Americans but not Caucasian Americans may underlie pathophysiologic differences in disease processes between races and may explain how African Americans accrue risk without the classical high-risk HLA types originally defined in Caucasians. Here we found that risk variants of two genes, *SH2B3* and *GAB3*, were more predictive of type 1 diabetes in African American subjects. Interestingly, both of these genes encode proteins that affect myeloid cell development and activation. *SH2B3* encodes the protein LNK, which modules cytokine signaling in myeloid cells via the signaling adapter, JAK2^37–41^. Indeed, the risk variant of *SH2B3*/Lnk (rs3184504) is associated with altered expression of key elements of IFNγ signaling^42^. Thus, it is likely that *SH2B3*/Lnk variants modulate myeloid innate immune cells through altered sensitivity to various cytokines. The *GAB3* protein product interacts with the M-CSF receptor and drives macrophage differentiation^43^. How the risk variant of *GAB3* affects this process remains unknown. As these functional studies advance, it will be critical that investigators consider the race of study subjects as well as the presence of additional gene variations that may affect the same cells/pathways.

An important aspect of our study assessed the effect of Hispanic/Latino ethnicity within southeastern U.S. Caucasians on GRS. We found that the GRS robustly discriminates type 1 diabetes patients from controls in Hispanic/Latino Caucasian to the same degree as non-Hispanic/Latino Caucasian cohorts. The prevalence of type 1 diabetes in Hispanic/Latino American youth is roughly half that of non-Hispanic/Latino Caucasians^34^, yet is increasing at a greater annual rate (4.2%) versus non-Hispanic/Latino Caucasian populations (1.2%)^44^. Given the concurrent increase in type 2 diabetes in non-Caucasian American youth^44^, our findings may have significant implications for utilization of GRS in both research and clinical settings in these understudied populations.

Although our cross-sectional cohort does not include routine follow-up, we were able to utilize GRS to identify type 1 diabetes subjects whose diagnoses were questionable or have been changed subsequent to their enrollment, which further demonstrates the clinical utility of GRS as a tool to improve differential diagnoses of type 1 diabetes from early-onset type 2 and monogenic diabetes^16,17^. This may justify the use of GRS as a screening tool at diagnosis in order to promote the concept of precision medicine when determining which therapies may be best suited for a particular patient. Notwithstanding, since GRS only modestly discerns type 1 diabetes patients from first-degree relatives, there may be a capacity to improve the current model. The log-additive model for non-HLA risk may not be the most accurate method for computing GRS, possibly resulting in decreased specificity or loss of age-associated non-HLA risk^45^. Additionally, there may be more comprehensive methods to capture all HLA-associated type 1 diabetes-risk with more HLA variants (reviewed in^1^). Since several loci contain genes that are predicted to confer overlapping functional effects (e.g., *CD25*, *IL2*, *PTPN2* in the IL-2 signaling pathway), one may expect a GRS model to include computations that account for such genetic synergies. However, this level of genetic risk modeling remains elusive, as Winkler *et al*. were unable to identify genetic interactions using a more extensive genotyping panel on a much larger cohort^15^. Moreover, models using genetics alone are not expected to predict type 1 diabetes with 100% accuracy since environmental, epigenetic, and stochastic factors (e.g., immunoreceptor V(D)J gene recombination) are also thought to impact overall risk. Perhaps even more confounding is the notion that genetic and environmental risk interactions may not be static phenomena. This may be most evident by the concomitant trends of decreasing proportion of high risk HLA in type 1 diabetes patients and increasing overall type 1 diabetes prevalence^46,47^. All of these aforementioned factors that are missing from this GRS model may contribute an unknown amount of bias negatively impacting GRS selectivity. Ultimately, improved accuracy of diabetes prediction models will likely require a better understanding of epistatic genetic and environmental risk interactions.

The results of this and other studies imply GRS could represent a low-cost means to assist in general population screening to identify patients who have increased risk of developing type 1 diabetes. We therefore envision the utilization of GRS to guide future trial recruitment and cohort stratification efforts. These observations strengthen the argument for focused genetic screening to monitor progression in the clinic, improve functional studies, facilitate biomarker identification, and optimize subject selection for interventional and natural history trials.

## Methods

### Subject enrollment and sample collection

Informed consent was obtained from subjects enrolled from outpatient clinics of the University of Florida, Gainesville, Florida; Nemours Children’s Hospital, Orlando, Florida; and Emory University, Atlanta, Georgia, under Institutional Review Board (IRB)-approval at each facility (IRB #201400709). All experiments were performed in accordance with relevant guidelines and regulations. Genomic DNA and serum samples were collected and stored at −20 °C from 1,946 research participants together termed the University of Florida Diabetes Institute (UFDI) cohort. This collection included control subjects [type 1 diabetes-unaffected and non-first- or -second-degree relatives of type 1 diabetes patients] (n = 405), first-degree relatives with ≤1 type 1 diabetes-relevant autoantibody (AAb) (n = 790), second-degree relatives with ≤1 AAb (n = 68), multiple AAb positive at-risk relatives (≥2AAb + , n = 46), and type 1 diabetes patients (n = 637). Type 1 diabetes status was assigned according to clinician diagnosis. Subjects self-reported race as Caucasian/White, African American/Black, Asian, Pacific Islander/Hawaiian, Native American/Alaskan, or Multiple/Other and separately indicated ethnicity as Hispanic/Latino or non-Hispanic/Latino (Supplemental Tables 1 and 2; Fig. 1A**)**. The geometric mean ± SD for the type 1 diabetes diagnosis age was 8.94 ± 2.18 years (Fig. 1B).

### AAb measurement

AAbs against type 1 diabetes-related autoantigens [i.e., glutamic acid decarboxylase (GAD), insulinoma-associated protein 2 (IA-2), and zinc transporter 8 (ZnT8)] were measured from serum samples via ELISA kits (KRONUS Inc., Star, ID) according to the manufacturer’s instructions^48^.

### DNA preparation

DNA was prepared via QiaCube high-throughput nucleic acid purification system according to manufacturer’s recommendations (Qiagen, Hilden, Germany). Purified DNA was genotyped on either the custom array or manually, as described below. Samples missing HLA SNP calls or with <90% of non-HLA risk measured were excluded. The OR of the type 1 diabetes-risk alleles were derived from Immunobase.org (Supplemental Table 3, Fig. 1D).

### Imputing HLA-DR-DQ diplotypes

HLA was imputed and odds ratios (OR) were computed as previously described^16,17^ (Fig. 1C; Supplemental Table 3). Following HLA imputation, samples were assigned one of six HLA diplotype categories: DR3-DQ2/DR4-DQ8, DR4-DQ8/DR4-DQ8, DR3-DQ2/DR3-DQ2, DR4-DQ8/X, DR3-DQ2/X, X/X, where X = non-DR3-DQ2 or non-DR4-DQ8. In addition, the highly protective HLA-DR15DQ6 haplotype was imputed, as well as HLA class-I A24 and B57, which were shown to confer susceptibility and protection, respectively, when conditioned on HLA class-II^1,49^.

### Single nucleotide polymorphism (SNP) selection and genotyping

The HLA-DR and HLA-DQ region plus additional loci with known associations for type 1 diabetes-risk^2^ were considered for inclusion in a custom Taqman SNP genotyping array (ThermoFisher, Carlsbad, CA). Since the list of risk loci changes as more GWAS and meta-analyses are completed, the loci in this study are limited to those that are curated on immunobase.org as of October 2017. SNP assays passed quality control (QC) when they generated >95% successful call rates and <5% intra-sample discordance. SNPs that failed QC were excluded. Some key SNPs that either failed QC on the array or were not included on the array (rs2187668, rs7454108, rs3129889, rs1264813, rs2395029, and rs2292239) were manually genotyped using validated Taqman assays (ThermoFisher, Carlsbad, CA)^16^. 32 SNPs passed QC (Supplemental Table 3**)**. The Taqman genotyping array and individual taqman assays were performed according to manufacturer instructions.

### Calculating GRS

The GRS calculation emulates a previously reported multivariate logistic regression model employed by Oram *et al*. and Patel *et al*.^16,17^:

$${\rm{GRS}}=\frac{({\sum }_{i=1}^{n}{\beta }_{i}{s}_{i})+{H}_{l}}{(n+1)\times 2}$$

where β is the natural log of the OR and *s* is the number of risk alleles (0, 1, or 2) carried for SNP *i* of *n* loci tested. Chromosome X SNPs in male subjects were counted as 0 or 2, which assumes a dominant risk effect in the hemizygous state. *H*_*l*_ is the HLA diplotype risk for combinations of DR3-DQ2, DR4-DQ8, and X. The summed risk was then divided by the number of alleles tested. This method used identical SNP imputing for class I and class II HLA as Oram *et al*. and Patel *et al*., and a partially overlapping set SNPs to compute non-HLA risk (compare Supplemental Table 3 to Oram *et al*.^16^).

### Statistics

Data were graphed and analyses performed using GraphPad Prism software version 7 (San Diego, CA). Data are presented as ROC curve with AUC, as Tukey box and whisker plots or mean ± SD bar graphs compared via Kruskal-Wallis with Dunn’s multiple comparisons testing, scatter plots with linear regression and Pearson Correlation, or in tabular form and compared via Fisher’s exact test. Fisher’s exact test was performed using the Scipy package (version 0.18.1, https://scipy.org/) in Python3. Balanced accuracy was calculated for thresholds across the GRS range as [(*predicted T1D*/*actual T1D*) + (*predicted non-T1D*/*actual non-T1D*)]/2. Significance was defined as *P* < 0.05.

## Electronic supplementary material

Supplemental Data
