# Supplementary material for: Application of a Genetic Risk Score to Racially Diverse Type 1 Diabetes Populations Demonstrates the Need for Diversity in Risk-Modeling
Source: Sci Rep. 2018 Mar 14;8:4529. doi: 10.1038/s41598-018-22574-5 (PMC5852207; doi:10.1038/s41598-018-22574-5)
Supplement: Supplementary file 1 — Supplemental Data [file 41598_2018_22574_MOESM1_ESM.pdf]

Application of a Genetic Risk Score to Racially Diverse Type 1 Diabetes Populations Demonstrates the Need for Diversity in Risk-Modeling

Daniel J. Perry, Clive H. Wasserfall, Richard A. Oram, MacKenzie D. Williams, Amanda Posgai, Andrew B Muir, Michael J. Haller, Desmond A. Schatz, Mark A. Wallet, Clayton E. Mathews, Mark A. Atkinson, Todd M. Brusko

Supplemental Figures

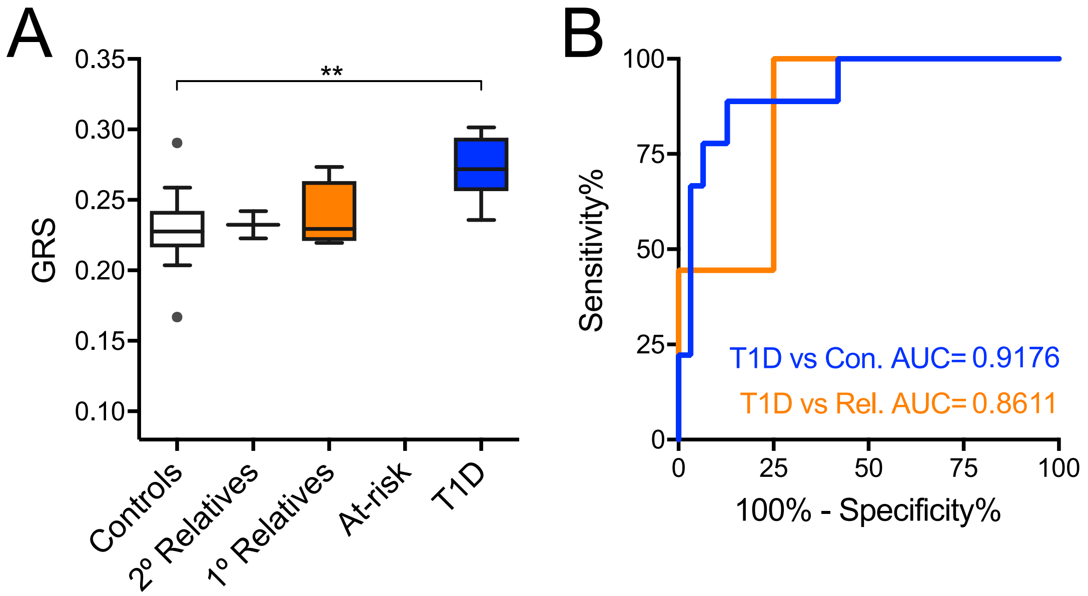

Figure S1. GRS discriminates Asian American (ASN) subjects with type 1 diabetes and high-risk relatives from controls and lower-risk relatives. (A) GRS was higher among type 1 diabetes patients (T1D, n=9) compared to controls (n=33), second-degree relatives (2° Relatives, n=2), and first-degree relatives (1° Relatives, n=4). Kruskal-Wallis ANOVA with Dunn's posttest ***P*<0.01. (B) Receiver operating characteristic (ROC) curve shows that the GRS discriminates T1D patients from control subjects (Con.) with 88.89% sensitivity yielding 80.65% specificity (area under curve (AUC)=0.9176) and T1D patients from first-degree relatives (Rel.) with 88.89% sensitivity yielding 75.00% specificity (AUC=0.8611).

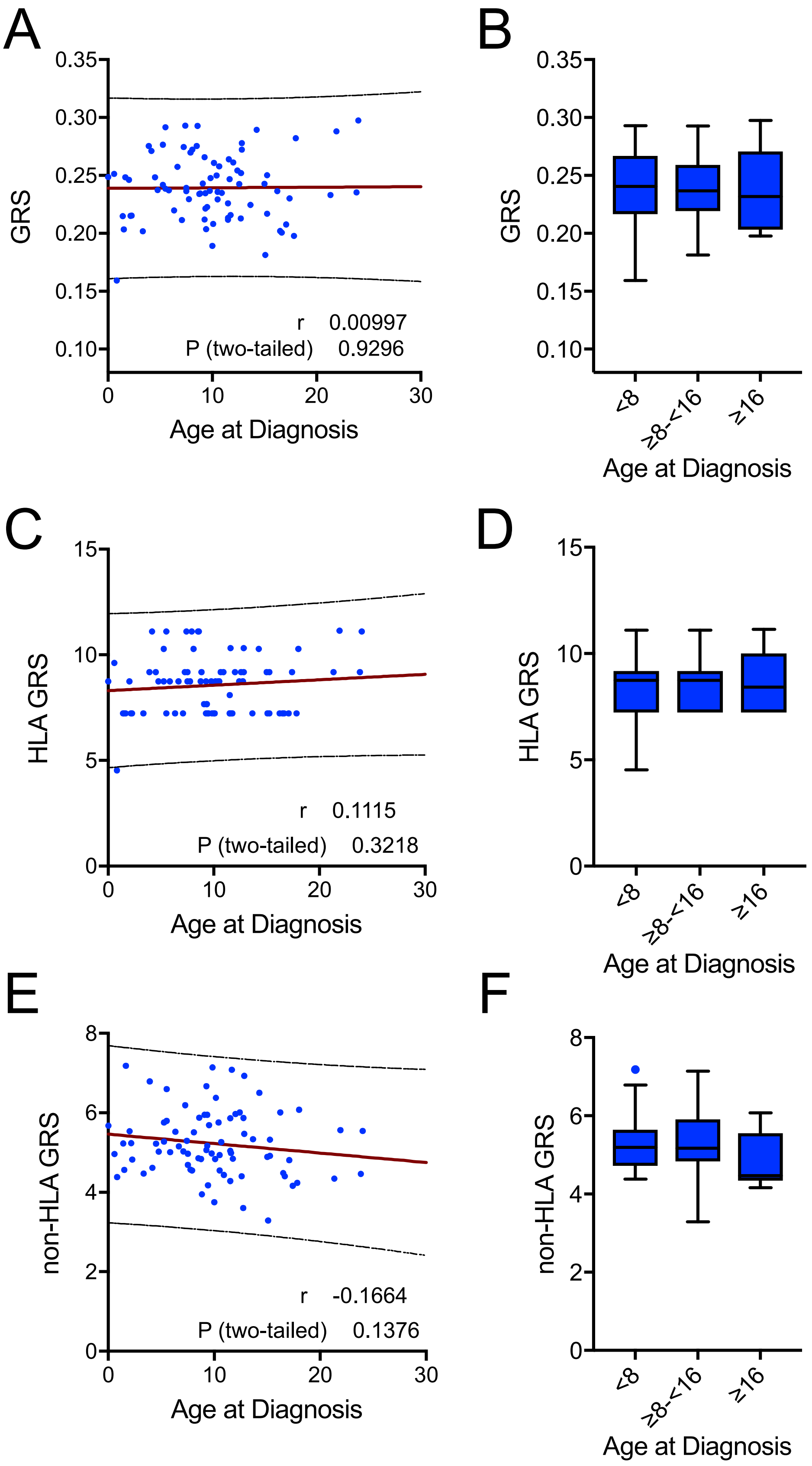

A
B
C
D
E
F
GRS
HLA GRS
non-HLA GRS
Age at Diagnosis
r 0.00997
P (two-tailed) 0.9296
r 0.1115
P (two-tailed) 0.3218
r -0.1664
P (two-tailed) 0.1376
<8
≥8-<16
≥16

Figure S2. HLA risk does not impart a genetic association with age of disease onset in the African American (AFR) subset of our cohort. (A) In AFR subjects, the genetic risk score (GRS) did not significantly correlate with age at diagnosis (linear regression analysis and Pearson correlation coefficient, *P*=0.93, r=0.0100). (B) GRS was not significantly different in AFR patients when grouped into under 8, 8-16, and over 16 years old at diagnosis (Kruskal-Wallis ANOVA with Dunn's posttest. (C-D) The HLA-only GRS imparted a similar lack of association with age at diagnosis as the full score in AFR patients (C: linear regression analysis and Pearson correlation coefficient, *P*=0.32, r=0.1115; D: Kruskal-Wallis ANOVA with Dunn's posttest. (E-F) The non-HLA GRS showed a weak trend toward negative correlation with age at diagnosis in AFR patients (E: linear regression analysis and Pearson correlation coefficient, *P*=0.14, r=-0.1667; F: Kruskal-Wallis ANOVA with Dunn's posttest *P*>0.05). The 99% probability bands for linear regressions are depicted as dotted lines.

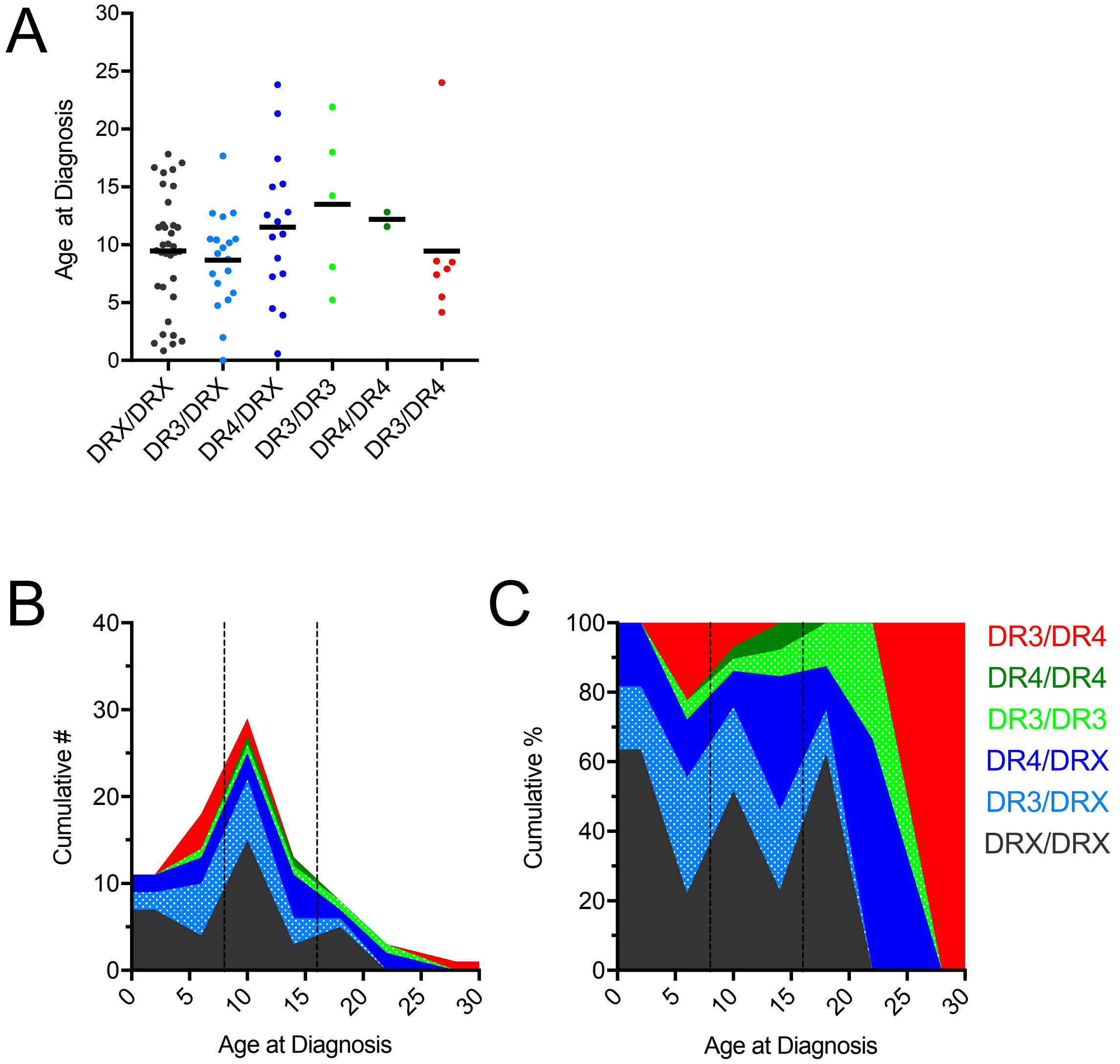

Figure S3. HLA versus Age at Diagnosis for the African American (AFR) Cohort Subset. (A) Age at diagnosis was not significantly different among patients grouped by HLA diplotype (Kruskal-Wallis ANOVA with Dunn's posttest *P*>0.05). (B) Stacked histogram depicting the cumulative number of patients grouped by HLA type versus their ages at diagnosis (4 year binned). (C) Stacked histogram depicting the cumulative percent of patients grouped by HLA type versus their ages at diagnosis (4 year binned). (B-C)

HLA-type is indicated by color as shown within the figure, and 8-year and 16-year age cutoffs are indicated by dashed lines.

Supplemental Tables

Supplemental Table 1. Demographic information for the University of Florida Diabetes Institute (UFDI) cohort. Sample size (n), sex distribution (percent male), and age (years) median (interquartile range) are reported for Caucasian (CAU), African American (AFR), Asian (ASN), and Other (multiple ethnicity [n=49], ethnicity not disclosed [n=93], Pacific Islander/Hawaiian [n=5], Native American/Alaskan [n=5]) subjects within the no-diabetes control subjects, second degree (2°) relatives, first degree (1°) relatives, multiple autoantibody positive (at-risk) subjects, and type 1 diabetes (T1D) patient cohorts as well as all enrolled subjects regardless of disease status (Totals).

Disease Status	CAU			AFR			ASN			Other			Total		
	n	% Male	Age	n	% Male	Age	n	% Male	Age	n	% Male	Age	n	% Male	Age
Controls	290	52.07	23.8 (16.6-33.1)	63	34.92	27.1 (16.0-35.7)	33	48.48	22.7 (17.7-27.1)	19	42.11	25.5 (15.8-36.4)	405	48.64	23.9 (16.4-33.3)
2º Relatives	33	36.36	18.0 (11.3-43.7)	28	21.43	33.5 (15.8-40.1)	2	50.00	24.5 (20.4-28.6)	5	20.00	49.0 (12.3-52.4)	68	29.41	26.8 (12.3-45.0)
1º Relatives	611	40.92	26.0 (11.6-41.8)	118	34.75	16.7 (10.5-33.9)	4	50.00	29.0 (16.6-40.7)	57	36.84	17.3 (10.8-37.3)	790	39.75	20.8 (11.3-40.4)
At-Risk	35	60.00	15.4 (10.9-34.4)	6	16.67	24.4 (13.6-33.7)	0	ND	ND	5	60.00	10.1 (8.2-14.3)	46	54.35	15.3 (10.3-33.8)
T1D	478	52.51	16.2 (12.1-22.0)	84	36.90	15.3 (12.4-17.6)	9	66.67	15.1 (13.4-19.0)	66	40.91	10.5 (5.7-14.9)	637	49.53	15.5 (11.7-19.8)
Totals	1447	47.34	18.4 (12.6-36.2)	299	33.78	16.9 (12.2-33.2)	48	52.08	20.3 (16.3-27.7)	152	39.47	14.0 (9.4-27.4)	1946	44.78	18.0 (12.3-34.8)

Supplemental Table 2. Demographic information for UFDI Hispanic cohort. Sample size (n), sex distribution (percent male), and age (years) median (interquartile range) are reported for Hispanic Caucasian (CAU), Hispanic African American (AFR), and multiple ethnicity or ethnicity not disclosed (Mul or ND) subjects within the no-diabetes control subjects, second degree (2°) relatives, first degree (1°) relatives, autoantibody positive (at-risk) subjects, and type 1 diabetes (T1D) patient cohorts. ND indicates no data (n=0).

Disease Status	CAU			AFR			Mul or ND		
	n	% Male	Age	n	% Male	Age	n	% Male	Age
Controls	37	59.46	21.3 (14.6-31.6)	1	100.00	42.0 (42.0-42.0)	5	20.00	33.4 (24.9-36.4)
2º Relatives	4	50.00	35.0 (27.9-43.6)	0	ND	ND	3	0.00	49.0 (29.3-55.3)
1º Relatives	104	44.23	17.0 (10.2-38.4)	3	33.33	10.6 (9.8-14.8)	22	36.36	34.8 (13.9-44.9)
At-Risk	5	40.00	18.2 (17.3-33.8)	1	0.00	15.5 (15.5-15.5)	2	100.00	8.2 (8.2-8.2)
T1D	45	46.67	15.6 (11.6-19.3)	4	25.00	10.7 (10.2-12.1)	16	37.50	15.0 (12.7-17.5)
Totals	195	47.69	17.1 (10.7-34.5)	9	33.33	10.9 (10.4-15.6)	48	35.42	18.3 (12.8-38.2)

Supplemental Table 3. Single nucleotide polymorphisms (SNPs) included in genetic risk score (GRS) calculations. The locus within the human genome is noted for each SNP along with associated candidate gene(s), risk allele on the forward strand (FWD), odds ratio (OR) for type 1 diabetes, p-value, and references.

Locus	SNP	Candidate Gene(s)	Risk Allele (FWD)	OR	p-value	Reference
6p21.32	rs2187668 rs7454108	DR3-DQ2 DR4-DQ8 diplotypes	DR3/4	48.18	ND	(9)
			DR4/4	21.98	ND	(9)
			DR3/3	21.12	ND	(9)
			DR4/X	7.03	ND	(9)
			DR3/X	4.53	ND	(9)
			DRX/X	1.00	ND	(9)
6p21.32	rs3129889	DR15-DQ6 haplotype	X	14.88	ND	(9)
6p21.32	rs1264813	A24 haplotype	A24	1.54	ND	(9)
6p21.32	rs2395029	B57 haplotype	X	2.50	ND	(8; 9)
1p13.2	rs2476601	PTPN22	A	1.89	1.1E-122	(42)
1q32.1	rs3024505	IL10	G	1.16	6.4E-08	(42)
2q24.2	rs1990760	IFIH1	T	1.17	5.84E-17	(42)
2q33.2	rs3087243	CTLA4	G	1.19	7.4E-21	(42)
3p21.31	rs11711054	CCR5	A	1.18	4.6E-08	(42)
4q27	rs17388568	ADAD1 IL2 IL21	A	1.13	1.06E-09	(42)
6q15	rs11755527	BACH2	G	1.13	5.4E-08	(43)
6q25.3	rs1738074	TAGAP	C	1.09	6.0E-03	(44)
9p24.2	rs1574285	GLIS3	G	1.12	1.03E-09	(42)
10p15.1	rs12722495	IL2RA	T	1.6	2.84E-39	(42)
10p15.1	rs2104286	IL2RA	T	1.24	5.98E-23	(42)
11p15.5	rs689	INS INS-IGF2 TH	T	2.39	1.11E-233	(42)
12q13.2	rs2292239	ERBB3	T	1.3	6.46E-19	(45)
12q14.1	rs10877012	CYP27B1	G	1.21	9.6E−04	(46)
12q24.12	rs653178	ATXN2 SH2B3 NAA25	C	1.3	1.56E-44	(42)
15q25.1	rs3825932	CTSH	C	1.16	1.16E-07	(43)
16p11.2	rs4788084	NUPR1 IL27	C	1.12	1.03E-09	(42)
16q23.1	rs7202877	CTRB2 CTRB1	G	1.28	3.1E-15	(47)
17q12	rs2290400	GSDMB ORMDL3	C	1.15	5.5E-13	(47)
17q21.2	rs7221109	CCR7 SMARCE1	T	1.05	1.3E-09	(47)
18p11.21	rs1893217	PTPN2	G	1.21	1.2E-15	(42)
18q22.2	rs763361	CD226	T	1.12	8.03E-11	(42)
19p13.2	rs2304256	TYK2	C	1.16	4.13E-09	(48)
19q13.33	rs602662	FUT2	A	1.12	4.23E-05	(49)
20p13	rs2281808	SIRPG SIRPB1	C	1.11	1.2E-11	(47)
21q22.3	rs11203203	UBASH3A	A	1.16	2.91E-15	(42)
22q12.3	rs229541	RAC2	G	1.11	1.8E-08	(42)
Xq28	rs2664170	GAB3 X	G	1.16	7.8E-09	(47)

Supplemental Table 4. Comparison of the genetic risk score (GRS) of the University of Florida Diabetes Institute (UFDI) cohort to the Wellcome Trust Case Control Consortium (WTCCC). GRS mean, standard deviation (SD), and 95% confidence interval (CI) are shown for Caucasian control and type 1 diabetes (T1D) subjects from the UFDI cohort as a comparison to the previously reported GRS of the WTCCC as calculated by Oram et al. and Patel et al.

Group	GRS of the UFDI Cohort		GRS of the WTCCC		References
	(Mean ± SD)	(95% CI)	(Mean ± SD)	(95% CI)	
Controls	0.231±0.03	0.226-0.235	0.229	0.228-0.230	(10)
T1D	0.277±0.03	0.274-0.279	0.279±0.03	0.278-0.280	(9; 10)

Supplemental Table 5. HLA versus Age at Diagnosis for the African American (AFR) Cohort Subset. The proportion of patients diagnosed age <8, age ≥8 to <16, and age ≥16 years within each HLA category is reported as number (N) and as odds ratio (OR). Fisher's exact test was used to determine if age at diagnosis was significantly different for each HLA type (*P*>0.05 all).

Age at Diagnosis	DRX/DRX		DR3/DRX		DR4/DRX		DR3/DR3		DR4/DR4		DR3/DR4	
	N	(OR)	N	(OR)	N	(OR)	N	(OR)	N	(OR)	N	(OR)
**<8**	11	(0.82)	8	(1.49)	5	(0.81)	1	(0.45)	0	(0.00)	4	(2.72)
**8-16**	18	(1.17)	10	(1.11)	8	(0.97)	2	(0.63)	2	(n/a)	2	(0.36)
**>16**	5	(1.03)	1	(0.27)	3	(1.49)	2	(4.53)	0	(0.00)	1	(0.98)
